# Cannabinoid CB_1_ Receptors Inhibit Gut-Brain Satiation Signaling in Diet-Induced Obesity

**DOI:** 10.3389/fphys.2019.00704

**Published:** 2019-06-11

**Authors:** Donovan A. Argueta, Pedro A. Perez, Alexandros Makriyannis, Nicholas V. DiPatrizio

**Affiliations:** ^1^ Division of Biomedical Sciences, School of Medicine, University of California, Riverside, Riverside, CA, United States; ^2^ Center for Drug Discovery, Northeastern University, Boston, MA, United States

**Keywords:** CB_1_R, cholecystokinin, enteroendocrine cell, gut-brain, obesity, satiation

## Abstract

Gut-brain signaling controls feeding behavior and energy homeostasis; however, the underlying molecular mechanisms and impact of diet-induced obesity (DIO) on these pathways are poorly defined. We tested the hypothesis that elevated endocannabinoid activity at cannabinoid CB_1_ receptor (CB_1_Rs) in the gut of mice rendered DIO by chronic access to a high fat and sucrose diet for 60 days inhibits nutrient-induced release of satiation peptides and promotes overeating. Immunoreactivity for CB_1_Rs was present in enteroendocrine cells in the mouse’s upper small-intestinal epithelium that produce and secrete the satiation peptide, cholecystokinin (CCK), and expression of mRNA for CB_1_Rs was greater in these cells when compared to non-CCK producing cells. Oral gavage of corn oil increased levels of bioactive CCK (CCK-8) in plasma from mice fed a low fat no-sucrose diet. Pretreatment with the cannabinoid receptor agonist, WIN55,212-2, blocked this response, which was reversed by co-administration with the peripherally-restricted CB_1_R neutral antagonist, AM6545. Furthermore, monoacylglycerol metabolic enzyme function was dysregulated in the upper small-intestinal epithelium from DIO mice, which was met with increased levels of a variety of monoacylglycerols including the endocannabinoid, 2-arachidonoyl-*sn*-glycerol. Corn oil failed to affect levels of CCK in DIO mouse plasma; however, pretreatment with AM6545 restored the ability for corn oil to stimulate increases in levels of CCK, which suggests that elevated endocannabinoid signaling at small intestinal CB_1_Rs in DIO mice inhibits nutrient-induced CCK release. Moreover, the hypophagic effect of AM6545 in DIO mice was reversed by co-administration with the CCK_A_ receptor antagonist, devazepide. Collectively, these results provide evidence that hyperphagia associated with DIO is driven by a mechanism that includes CB_1_R-mediated inhibition of gut-brain satiation signaling.

## Introduction

Food intake and energy homeostasis are controlled by a dynamic interplay of gut-brain signaling pathways that are not well-defined but are thought to become dysregulated in obesity (Steinert et al., 2017). Recent studies in humans and rodents suggest a critical role for the endocannabinoid (eCB) system in these processes (DiPatrizio and Piomelli, 2012, 2015; DiPatrizio, 2016). The eCB system is located in cells throughout the body and is comprised of the eCBs, 2-arachidonoyl-*sn*-glycerol (2-AG) and anandamide (AEA), their biosynthetic and degradative enzymes, and the cannabinoid receptor subtypes 1 and 2 [CB_1_R and CB_2_R, respectively (Piomelli, 2003; Pertwee, 2015)]. CB_1_Rs in the brain control food intake and energy homeostasis (DiPatrizio and Piomelli, 2012; Simon and Cota, 2017); however, targeting central CB_1_Rs with antagonists for the treatment of human obesity led to psychiatric side-effects that preclude their use as safe and effective anti-obesity therapeutics (Christensen et al., 2007). In contrast, CB_1_Rs antagonists that are restricted to the periphery and do not readily cross the blood-brain barrier are associated with improvements in a variety of metabolic parameters in rodents, and may be an effective anti-obesity strategy that is devoid of psychiatric side-effects inherent to brain-permeable drugs (LoVerme et al., 2009; Randall et al., 2010; Cluny et al., 2011; DiPatrizio et al., 2011, 2013, 2015; Tam et al., 2012, 2017; Maccarrone et al., 2015). Nonetheless, peripheral mechanisms influence brain function [e.g., signals from the gut microbiome (Cani and Knauf, 2016)]; thus, the impact of disrupting endocannabinoid signaling at peripheral CB_1_Rs on these functions is largely unknown and warrants future investigation.

Studies from our lab and others suggest key roles for the peripheral eCB system in controlling feeding behavior and energy homeostasis (DiPatrizio, 2016; Hillard, 2017; Simon and Cota, 2017). Indeed, eCB levels are increased in the small intestinal epithelium of rodents (1) during a fast (Gomez et al., 2002; Izzo et al., 2009; DiPatrizio et al., 2015; Argueta and DiPatrizio, 2017), (2) after oral exposure to dietary fats (DiPatrizio et al., 2011, 2013), and (3) in a mouse model of western diet-induced obesity (DIO) (Argueta and DiPatrizio, 2017). Pharmacological inhibition of elevated eCB signaling at small-intestinal CB_1_Rs with peripherally-restricted CB_1_R antagonists blocks (1) re-feeding after a fast (DiPatrizio et al., 2015), (2) intake of dietary fats based on their orosensory properties (DiPatrizio et al., 2011, 2013), and (3) overeating (i.e., increased meal size and caloric intake) associated with DIO, (Argueta and DiPatrizio, 2017). These studies suggest that the eCB system in the small intestinal epithelium plays a key role in feeding behavior and energy balance, and becomes dysregulated in DIO.

The mechanism(s) underlying eCB control of gut-brain signaling and its dysregulation in DIO is largely unknown. Nonetheless, CB_1_Rs are expressed on the afferent vagus nerve and suggested to control feeding behavior and energy balance by directly modifying gut-brain vagal signaling important for food intake (Burdyga et al., 2004, 2006). For example, expression of CB_1_Rs in the rat nodose ganglion is upregulated after fasting, and refeeding or administration of the gut-derived satiation peptide, cholecystokinin (CCK), reversed this effect (Burdyga et al., 2004, 2010). Moreover, both, fasting-induced increases in CB_1_R expression in the nodose ganglion and the ability for CCK to decrease this response were blunted in rats fed a high-fat diet (Cluny et al., 2013). Vianna and colleagues, however, reported that select deletion of CB_1_Rs on the afferent and efferent vagus nerve had no effect on food intake or body weight in mice fed a standard rodent chow or high-fat diet (Vianna et al., 2012). These findings suggest that CB_1_Rs expressed on the vagus nerve may be dispensable for feeding behavior and maintenance of body weight.

Dietary fats and other macronutrients are sensed by enteroendocrine cells in the small intestinal epithelium and stimulate release of satiation peptides including CCK (McLaughlin et al., 1998, 1999; Raybould et al., 2006; Steinert et al., 2017), which controls meal size and satiation by activating CCK_A_ receptors on the afferent vagus nerve (Smith et al., 1981, 1985; Schwartz and Moran, 1994; Raybould, 2007; Dockray, 2013; Kaelberer et al., 2018; Schwartz, 2018) and possibly in the brain (Reidelberger et al., 2004; Ripken et al., 2015). Furthermore, CCK-containing I-cells in the upper small intestinal epithelium of mice express genes for CB_1_Rs (Sykaras et al., 2012). Thus, CB_1_Rs in the small intestinal epithelium may control feeding behavior by an indirect mechanism that includes controlling release of gut-derived satiation peptides. We investigated this possibility by testing the hypothesis that elevated endocannabinoid activity at CB_1_Rs in the gut of mice rendered DIO by chronic access to a high-fat and sucrose diet inhibits nutrient-induced release of satiation peptides, which in turn, leads to overeating by delaying satiation.

## Materials and Methods

### Animals

Eight-week old C57BL/6 mice (Taconic, Oxnard, CA, USA) were group-housed with *ad libitum* food and water access and maintained on a 12 h dark/light cycle. C57BL/6-Tg (Cck-EGFP)2Mirn/J mice with enhanced green fluorescent protein on the promoter for cholecystokinin were used for immunohistochemistry and fluorescence-activated cell sorting (FACS) of small intestinal CCK-containing cells (Jackson Laboratories, Bar Harbor, ME, USA). Test diets included Teklad 2020x soy-purified Standard Rodent Chow (SD; Envigo, Huntingdon, UK) or Western-style diet (WD; Research Diets D12709B, New Brunswick, NJ, USA; 40% kcal as fat, 43% kcal as carbohydrates, mainly sucrose). Body weights were recorded every other day at noon. To assess feeding behaviors, mice were single-housed in behavior chambers (TSE Systems, Chesterfield, MO, USA). All procedures met the U.S. National Institute of Health guidelines for care and use of laboratory animals and were approved by the Institutional Animal Care and Use Committee of the University of California, Riverside.

#### Feeding Behaviors

Animals were placed into feeding chambers 5 days prior to recording for acclimation, and testing began at 60 days after being placed on their respective experimental diets. Feeding behaviors were assessed starting 1 h prior to dark cycle (1,700 h) over a 24 h period for acclimation and for 12 h following drug administrations. Behavioral parameters include total caloric intake, average meal size, average rate of intake, average number of meals, first meal size, average meal duration, and average post meal interval. Data were processed using TSE Phenomaster software.

### Chemicals and Administration Schedule

AM6545, a peripherally-restricted CB_1_R neutral antagonist, was given by IP injection at 10 mg per kg (Northeastern University Center for Drug Discovery, Boston, MA, USA). Devazepide (Tocris, Bristol, UK), a CCK_A_ receptor antagonist, was given IP at 0.3 mg per kg. Both drugs were dissolved in vehicle consisting of 7.5% DMSO, 7.5% Tween80, and 85% sterile saline, and warmed in a water bath to ensure solubility. All control conditions were matched, using vehicle in place of drugs and injections occurred 1 h prior to behavior recording (1,600 h). A 72-h washout period was allowed between drug treatments. JZL184 (Tocris, Bristol, UK), a potent inhibitor of monoacylglycerol lipase (MGL), was used to prevent monoacylglycerol hydrolysis in the diacylglycerol lipase (DGL) assay and to validate our MGL assay (described below). Tetrahydrolipstatin (Tocris, Bristol, UK), a lipase inhibitor used routinely to study DGL activity (Gregg et al., 2012; Jung et al., 2012), was used to validate our DGL assay.

### Measurement of Intestinal Lipids

#### Tissue Harvest and Lipid Extraction

Animals were anesthetized with isoflurane at time of tissue harvest (1,500–1,700 h) following *ad libitum* food and water access. Blood was collected by cardiac puncture and deposited into vacutainers containing EDTA; plasma was collected as supernatant following 10 min centrifugation at 1,500 *g* (kept at 4°C). Jejunum was quickly removed and washed in phosphate-buffered saline (PBS), opened longitudinally on a stainless steel tray on ice, and contents were removed. Jejunum mucosa was isolated using glass slides to scrape the epithelial layer and was snap-frozen in liquid N_2_. Samples were stored at −80°C pending analysis. Frozen tissues were weighed and then homogenized in 1 ml methanol solution containing 500 pmol [^2^H_5_]-2-AG (Cayman Chemicals, Ann Arbor, MI) as an internal standard. Lipids were extracted as previously described (Argueta and DiPatrizio, 2017) and resuspended in 0.1 ml methanol:chloroform (9:1) and analyzed *via* ultra-performance liquid chromatography tandem mass spectrometry (UPLC-MS/MS).

#### LCMS Detection of 2-Arachidonoyl-*sn*-Glycerol and Other Monoacylglycerols

Data were acquired using an Acquity I Class UPLC with direct connection to a Xevo TQ-S Micro Mass Spectrometer (Waters Corporation, Milford, MA, USA) with electrospray ionization (ESI) sample delivery. Lipids were separated using an Acquity UPLC BEH C_18_ column (2.1 mm × 50 mm i.d., 1.7 μm, Waters Corporation) and inline Acquity guard column (UPLC BEH C_18_ VanGuard PreColumn; 2.1 mm × 5 mm i.d.; 1.7 μm, Waters Corporation), and eluted by a gradient of water and methanol (containing 0.25% acetic acid, 5 mM ammonium acetate) at a flow rate of 0.4 ml per min and gradient: 80% methanol 0.5 min, 80 to 100% methanol 0.5–2.5 min, 100% methanol 2.5–4.5 min, 100 to 80% methanol 4.5–4.6 min, and 80% methanol 4.6–5.5 min. The column was maintained at 40°C, and samples were kept at 10°C in accompanying sample manager. MS/MS detection was in positive ion mode with capillary voltage maintained at 1.10 kV, and argon (99.998%) was used as collision gas. Cone voltages and collision energies for respective analytes: 2-AG (20:4) = 30v, 12v; 2-DG (22:6) = 34v, 14v; 2-PG (16:0) = 18v, 10v; 2-OG (18:1) = 42v, 10v; 2-LG (18:2) = 30v, 10v; monononadecadienoin (19:2 monoacylglycerol; product of DGL assay, see below in “DGL Activity Assay”) = 18v, 10v; and [^2^H_5_]-2-AG = 25v, 44v. Lipids were quantified using a stable isotope dilution method detecting H^+^ or Na^+^ adducts of the molecular ions [M + H/Na]^+^ in multiple reaction monitoring (MRM) mode. Acyl migration occurs in monoacylglycerols; thus, the sum of 2-AG and 1-AG is presented. Tissue processing and LCMS analyses for experiments occurred independently of other experiments. Extracted ion chromatograms for MRM transitions were used to quantify analytes: 2-AG (*m/z* = 379.3 > 287.3), 2-DG (*m/z* = 403.3 > 311.1), 2-PG (*m/z* = 331.3 > 239.3), 2-OG (*m/z* = 357.4 > 265.2), 2-LG (*m/z* = 355.3 > 263.3), 19:2 monoacylglycerol (*m/z* = 386.4 > 277.2), and [^2^H_5_]-2-AG (*m/z* = 384.3 > 93.4), which was used as an internal standard for quantitation of monoacylglycerols.

### ELISA Analysis of CCK-8 Octapeptide

Mice were fasted for 12 h in order to ensure an empty stomach. Mice were pretreated with CB_1_R ligands, then administered corn oil (0.5 ml) by oral gavage 30 min later. Levels of CCK-8 were assessed in blood plasma 30 min following gavage. Blood was placed in BD vacutainer lavender-top EDTA blood collection tubes on ice and plasma obtained by centrifugation of tubes at 1,500 *g* for 10 min at 4°C by a sensitive and selective CCK-8 ELISA (Cloud Clone Corp; Katy, TX, USA). Mice were maintained for 60 days on standard diet (SD) and given IP injection of vehicle or the general cannabinoid receptor agonist, WIN55,212-2 (3 mg per kg), or WIN 55,212-2 in combination with the peripherally-restricted CB_1_R antagonist, AM6545 (10 mg per kg). In addition, mice maintained for 60 days on Western diet (WD) were given IP injection of vehicle or AM6545 (10 mg per kg). ELISA reaction was measured using iMark microplate reader (BioRad, Hercules, CA, USA).

### Immunohistochemistry

Intact proximal small intestine was removed, and contents were flushed with ice-cold 4% paraformaldehyde (PFA)/PBS, then fixed in 4% PFA for 4 h at 4°C. Samples were transferred to 20% sucrose/PBS and incubated for 1 day at 4°C for cryopreservation. Cross sections of upper small intestine were cut and frozen in OCT (Fisher Healthcare, Chino, CA, USA) on dry ice. About 16 μm sections were taken on a cryostat (Leica) and mounted onto charged glass slides. Sections were permeabilized with 0.5% Tween-20/PBS and then blocked with 0.1% Tween in casein solution (Thermo Fisher). Primary antibodies from rabbit for Cholecystokinin (CCK; ABcam, Cambridge, UK) and Cannabinoid Receptor 1 (Generously provided by Dr. Ken Mackie, Indiana University) were diluted 1:500 in blocking buffer and separately added to slides. Slides were washed three times with 0.1% Tween/PBS solution before being incubated with AlexaFluor 647 (Donkey anti-rabbit, Thermo Fisher). Tissue was washed again and mounted with Prolong Gold Antifade reagent with DAPI (Thermo Fisher) for nuclear counterstaining. Images were obtained at room temperature using an Axio Observer Z1 Inverted Microscope (Zeiss, Oberkochen, Germany) at 63× magnification with a CSU-X1 Confocal Scanner Unit (Yokogawa, Tokyo, Japan), and images were captured using a Prime 95B Scientific CMOS Camera (Photometrics, Huntington Beach, CA, USA). Micro-Manager open source software was used for image capture, and final images were optimized using ImageJ 1.51n (NIH, Bethesda, MD, USA).

### Fluorescence-Activated Cell Sorting

#### Isolation of Intestinal Epithelial Cells

Approximately, 4 cm of proximal small intestine was inverted and mechanically disrupted with frosted glass slides into ice-cold buffer containing 5% BSA, 0.6 mM dithiothreitol (DTT) and 1 mM EDTA in PBS to disrupt mucosal cell layer. Live cells were counted following trypan blue staining and 20 × 10^6^ cells were pelleted at 200 *g* for 5 min and resuspended in 1 ml of 3% BSA containing 1 mM EDTA in PBS. Cell suspension was filtered through 30-micron mesh and subsequently processed by fluorescence-activated cell sorting (FACS).

#### FACS Sorting of eGFP (+) and eGFP (−) Cells

**Figure 1:**
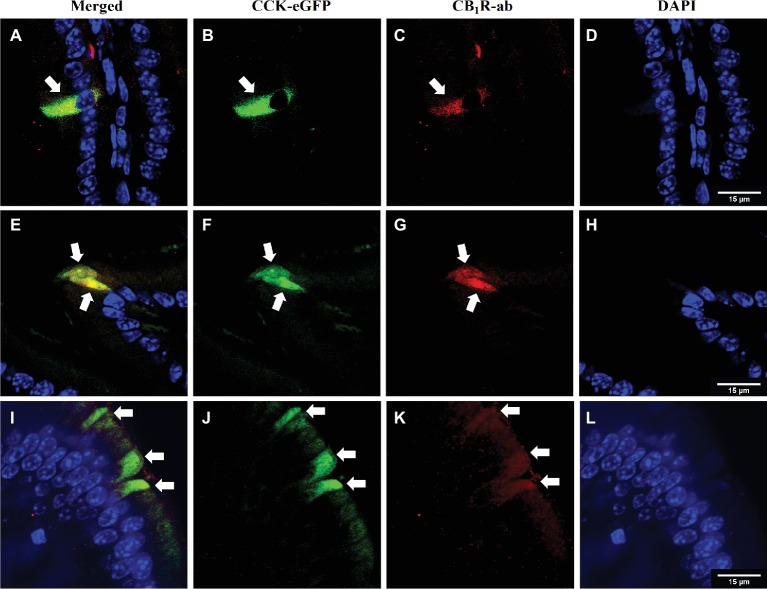
CB1Rs co-localize with CCK-containing cells in the upper small-intestinal epithelium. Immunohistochemical detection of eGFP [CCK-eGFP (B, F, J)] and CB_1_Rs [CB_1_R-ab **(C, G, K)**] reveals co-localization [merge **(A, E, I)**] in villi of intestinal epithelium. Arrows indicate separate enteroendocrine cells that contain immunoreactivity for CB_1_Rs that co-localize with CCK-eGFP cells. Representative images from three CCK-eGFP mice [DAPI stain **(D,H,L)**].

Isolated cells were sorted and analyzed on a MoFlo Astrios (Beckman Coulter, Brea, CA, USA). Debris was detected and excluded using forward and side scatter profiles generated with a 488 nm laser. eGFP positive (+) cells were detected by fluorescence intensity, using excitation and emission spectra of 488 and 513/26, respectively. A wild-type mouse from C57Bl/6 J background was used to establish autofluorescence, and gating for eGFP was used for final sorting (See Figures 1D,E). Samples were sorted into fresh resuspension buffer prior to qPCR analysis of gene expression. Mice were fasted for 10 h prior to acquisition of cells.

### Enzyme Activity Assays

#### Tissue Preparation

Intestinal epithelium was collected as described above (2.3.1) and approximately 100 mg of frozen tissue was homogenized in 2 ml of ice-cold 50 mM Tris-HCl, 320 mM sucrose (pH 7.5) buffer. Homogenates were centrifuged at 800 *g* for 10 min at 4°C and supernatant was collected. Protein supernatants were sonicated twice for 10 s and then freeze-thawed in liquid nitrogen twice. Samples were spun again, and supernatant protein content was quantified using BCA assay and diluted to working concentration with Tris-HCl/sucrose buffer.

#### DGL Activity Assay

Small-intestinal epithelial tissue homogenates (25 μg, room temperature) were incubated with the MGL inhibitor, JZL184 (0.3 μM), for 10 min in order to block MGL activity during the assay. Homogenates were then incubated in 0.2 ml solution of Tris-HCL with 0.2% Triton X-100 (pH 7.0) containing 20 nmol dinonadecadienoin (19:2 DAG) at 37°C for 30 min. Reactions were stopped by adding 1 ml of ice-cold MeOH containing 25 pmol [^2^H_5_]-2-AG as internal standard. Lipids were extracted and the product of the reaction, monononadecadienoin (19:2 monoacylglycerol), was analyzed *via* UPLC/MS/MS as described above for 19:2 monoacylglycerol (See “LCMS Detection of 2-Arachidonoyl-*sn*-Glycerol and Other Monoacylglycerols”).

#### MGL Activity Assay

Small-intestinal epithelial tissue (10 μg) was incubated with 0.4 ml solution of Tris-HCL with 0.1% BSA (pH 8.0) containing 50 nmol nonadecadienoin (19:2 monoacylglycerol; Nu-Chek Prep, Waterville, MN, USA; final volume 0.5 ml per reaction) at 37°C for 10 min. Reactions were stopped by adding 1 ml of MeOH containing 10 nmol heptadecanoic acid (17:1 FFA; Nu-Chek Prep) as internal standard. Lipids were extracted and the product of the reaction (19:2 free fatty acid) was analyzed *via* UPLC/MS/MS according to the following protocol. Data were acquired using equipment described above (See “LCMS Detection of 2-Arachidonoyl-*sn*-Glycerol and Other Monoacylglycerols”) and eluted by a gradient of water and methanol (containing 0.25% acetic acid, 5 mM ammonium acetate) at a flow rate of 0.4 ml per min and gradient: 90% methanol 0.1 min, 90–100% methanol 0.1–2.0 min, 100% methanol 2.0–2.1 min, 100 to 90% methanol 2.1–2.2 min, and 90% methanol 2.2–2.5 min. Column was maintained at 40°C and samples were kept at 10°C in sample manager. MS detection was in negative ion mode with capillary voltage maintained at 3.00 kV. Cone voltages for nonadecadienoic acid (19:2 FFA) = 48v and heptadecanoic acid (17:1 FFA) = 64v. Lipids were quantified using a stable isotope dilution method of proton adducts of the molecular ions [M–H]^−^ in selected ion recording (SIR) mode. Tissue processing and LCMS analyses for experiments occurred independently of other experiments. Extracted ion chromatograms for SIR masses were used to quantify analytes: 19:2 FFA (*m/z* = 293.2) product of MGL enzyme assay and 17:1 FFA (*m/z* = 267.2) as internal standard.

#### Gastric Emptying

To evaluate drug or endogenous endocannabinoid effects on gastric emptying, corn oil was spiked with 1.0 nmol 19:2 FFA and administered by oral gavage (500 μl), then quantities of 19:2 FFA remaining in the stomach were evaluated at the time of blood collection 30 min after gavage. The stomach was removed and immediately placed into methanol containing 17:1 FFA as internal standard. Lipids were extracted and 19:2 FFA was detected and quantified as above (See “MGL Activity Assay”).

### Gene Expression Analysis

#### RNA Isolation From Intestinal Epithelium

Total RNA was extracted from intestinal epithelium using RNeasy kit (Qiagen, Valencia, CA, USA) method, and first-strand complementary DNA was generated using M-MLV reverse transcriptase (Invitrogen, Carlsbad, CA, USA). All surfaces for tissue collection and processing were sanitized using 70% ethanol and then treated with an RNAse inhibitor (RNAse out, G-Biosciences, St. Louis, MO, USA) to maintain integrity of isolated RNA. Reverse transcription of total RNA (1 μg epithelium) was performed as previously described (Argueta and DiPatrizio, 2017).

#### RNA Isolation From Sorted Cells

Sorted cell suspensions were pelleted at 3,000 *g* for 10 min and resuspended in 0.5 ml of Qiazol (Qiagen, Valencia, CA) and subsequently processed using RNeasy kit to isolate total RNA. Reverse transcription was performed as described above using 50 ng total RNA.

#### Quantitative Polymerase Chain Reaction Analysis

RT-qPCR was carried out using PrimePCR Sybr Green Assays (Biorad, Hercules, CA, USA) with the following primers for mouse genes: CB_1_R (Cnr1), CB_2_R (Cnr2), cholecystokinin (Cck), fatty-acid amide hydrolase (Faah), n-acyl phosphatidyl ethanolamine-specific phospholipase D (Napepld), diacylglycerol lipase alpha (Dagla) and beta (Daglb), monoacylglycerol lipase (Mgll), alpha-beta-hydrolyzing domain 6 (Abhd6) with Hprt and Actb as housekeeping genes for epithelium and sorted cells, respectively. Values are expressed as relative mRNA expression based on widely used methods [i.e., delta-delta cq; see (Livak and Schmittgen, 2001)]. Reactions were run in triplicate for each animal.

### Statistical Analysis

Values are expressed as means ± SEM. Unpaired Student’s two-tailed *t*-test was used to compare data for standard diet- and western diet-fed groups. Repeated measures two-way ANOVA was used for groups measured over time. Additionally, regular one-way and two-way ANOVA were used to determine differences in multiple groups with *post-hoc* Sidak’s multiple comparisons tests or Newman-Keul’s, as appropriate. Data were analyzed using GraphPad Prism6 software. Significance was determined as *p* < 0.05. Statistical outliers were determined using Grubb’s test in all datasets.

## Results

### CB_1_Rs Are Expressed in CCK-Containing Cells in the Upper Small-Intestinal Epithelium

**Figure 2:**
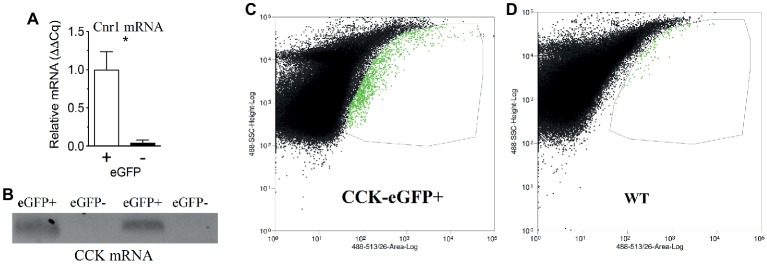
CB1R mRNA expression is enriched in CCK-containing cells in the upper small-intestinal epithelium. Fluorescence-activated cell sorting (FACS) of eGFP-CCK-positive (+) and eGFP-CCK-negative (−) cells from the upper small-intestinal epithelium reveals enhanced Cnr1 expression in eGFP-CCK-positive cells **(A)**. Expression of mRNA for CCK is found in eGFP-CCK-positive cells but not in eGFP-CCK-negative cells **(B)**. Gating strategy shown for sorting of eGFP-positive and eGFP-negative events, with eGFP-positive cells highlighted in green and demarked by thin line **(C)** and compared to upper small-intestinal epithelial cells from a wild-type (WT) mouse **(D)**. Data expressed as mean ± S.E.M. Analyzed using Student’s *t*-test, two-tailed **(C)**; *n* = 3 per group; *p < 0.05.

We reported that eCB levels are increased in the upper small-intestinal epithelium from mice maintained on a Western Diet (WD; high-fat and sucrose diet) for 60 days when compared to lean controls maintained on a low-fat and low-sugar diet, and inhibiting peripheral CB_1_Rs blocked overeating associated with consumption of WD (i.e., increased meal size, rate of food intake, and total caloric intake) (Argueta and DiPatrizio, 2017). To identify the molecular underpinnings of gut-brain eCB signaling important for feeding behavior and its dysregulation in DIO, we first evaluated whether CB_1_Rs are expressed in cells that produce and secrete the satiation peptide, CCK. CCK controls meal size and induces satiation by activating CCK_A_ receptors on the afferent vagus nerve (Smith et al., 1981, 1985; Schwartz and Moran, 1994; Reidelberger et al., 2004; Raybould, 2007; Dockray, 2013; Ripken et al., 2015). CB_1_R immunoreactivity was found in CCK-eGFP-positive cells from the upper small intestinal epithelium (Figure 1) in a mouse line that expresses eGFP selectively in CCK-expressing cells [C57BL/6-Tg(Cck-EGFP)2Mirn/J] (Schmidt et al., 2014). Furthermore, immunoreactivity for CCK was co-localized with eGFP in the upper small-intestinal epithelium, which confirms expression of CCK in eGFP-containing cells from this mouse line (Supplementary Figure S1). We, next isolated eGFP-positive and eGFP-negative cells from the upper small intestinal epithelium by fluorescence-activated cell sorting (FACS). Messenger RNA (mRNA) for CB_1_Rs (Cnr1) was enriched in CCK-eGFP-positive cells when compared to CCK-eGFP-negative cells (Figure 2A; eGFP-positive = 1.00 ± 0.24, eGFP-negative = 0.04 ± 0.04; *p* = 0.016; data from three mice). Moreover, mRNA for CCK was present in CCK-eGFP-positive cells isolated by FACS but was not present in CCK-eGFP-negative cells, which highlights the specificity of our FACS gating strategy for isolating CCK-eGFP cells and further confirms expression of CCK in these cells (Figure 2B). Our gating strategy was optimized for sorting of eGFP-positive and eGFP-negative events from cells isolated from the upper small-intestinal epithelium of CCK-eGFP mice (see Figure 2C). Cells from wild-type mice (see Figure 2D) show minimal fluorescence at less than 10% of levels found in CCK-eGFP cells: eGFP-positive cells comprise 0.63% of total cells analyzed from CCK-eGFP mice, and wild-type show 0.06%, likely due to autofluorescence (see Supplementary Figure S2 for detailed FACS report). These results suggest that CCK-containing I-cells in the mouse upper small-intestinal epithelium are enriched in expression of CB_1_Rs.

### Peripheral CB_1_Rs Control Fat-Induced CCK Secretion

**Figure 3:**
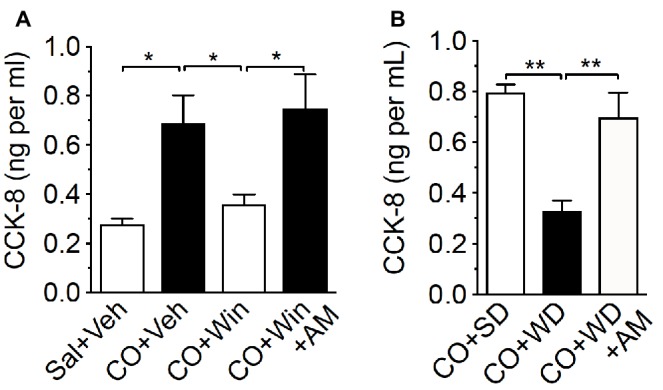
Exogenous or endogenous activation of peripheral CB1Rs inhibits fat-induced CCK release. Compared to control [0.5 ml saline (Sal) by oral gavage and vehicle (Veh) by IP injection], corn oil (CO; 0.5 ml by oral gavage) increased levels of CCK-8 in plasma of lean mice fed a low-fat no-sugar standard diet (SD), an effect blocked by the CB_1_R agonist, WIN 55,212-2 (WIN, IP 3 mg per kg 30 min before CO) **(A)**. The effects of WIN were inhibited by co-administration with the peripherally-restricted CB_1_R antagonist, AM6545 (AM; 10 mg per kg 30 min before CO). When compared to control SD mice (CO + SD), CO failed to elicit changes in levels of CCK-8 in plasma in mice fed western diet (WD) for 60 days, and inhibition of peripheral CB_1_Rs with AM6545 normalized levels of CCK-8 to those found in SD CO controls **(B)**. Data expressed as means ± S.E.M. Analyzed by one-way ANOVA with *post hoc* Newman-Keuls multiple comparison test. *n* = 3–5 per condition, * *p* < 0.05, ** *p* < 0.01.

The arrival of fat and other macronutrients into the duodenum stimulates release of a variety of signaling molecules that include CCK, which is produced and secreted by enteroendocrine I-cells lining the upper small-intestinal epithelium (McLaughlin et al., 1999; Raybould, 1999; Raybould et al., 2006; Cvijanovic et al., 2017; Steinert et al., 2017). We next tested the hypothesis that CB_1_Rs control nutrient-induced release of CCK from the upper small-intestinal epithelium. Oral gavage of corn oil (CO) in lean mice maintained on a standard rodent diet (SD; low-fat no-sucrose chow) increased plasma levels of bioactive CCK, CCK-8 (octapeptide), when compared to control mice that received oral gavage of saline [Figure 3A; CO = 0.69 ± 0.11 ng per ml, saline control = 0.28 ± 0.02 ng per ml; *p* < 0.05 CO versus saline control, *n* = 3–5). Peripheral administration of the general cannabinoid receptor agonist, WIN55,212-2 (WIN, 3 mg per kg), blocked CO-induced secretion of CCK-8 (Figure 3A; CO + WIN = 0.36 ± 0.04 ng per ml; *p* < 0.05 CO + WIN versus CO alone, *n* = 5). Furthermore, the effect of WIN administration on CO-induced secretion of CCK-8 was reversed by co-treatment with the peripherally-restricted neutral CB_1_R-selective antagonist, AM6545 (Figure 3A; CO + WIN+AM6545 = 0.75 ± 0.14 ng per ml; *p* < 0.05 CO + WIN versus CO + WIN+AM, *n* = 5; AM6545 10 mg per kg). These results suggest that exogenous activation of CB_1_Rs inhibits nutrient-induced CCK release from the upper gut.

We next tested the hypothesis that elevated endogenous activity (e.g., increased 2-AG levels) at upper small-intestinal CB_1_Rs in mice maintained on Western Diet (WD; high-fat and sucrose diet) for 60 days inhibits CO-induced increases in circulating levels of CCK-8. We first confirmed that levels of 2-AG—among other monoacylglycerols—were increased in the upper small intestinal epithelium of WD mice, when compared to lean mice fed SD for 60 days (see Table 1). Next, we tested the ability for oral gavage of CO to increase CCK-8 levels in plasma of WD mice. CO failed to affect levels of CCK-8 in WD mice when compared to mice fed a standard diet (SD) that is low in fat and absent of sucrose (Figure 3B; CO + WD = 0.33 ± 0.04 ng per ml, CO + SD = 0.8 ± 0.03 ng per ml; *p* < 0.01, *n* = 5). Furthermore, AM6545 treatment in WD mice that received oral gavage of CO increased levels of CCK-8 to those comparable to SD mice under the same conditions (Figure 3B; CO + WD + AM = 0.7 ± 0.1 ng per ml; *p* < 0.01 CO + WD versus CO + WD + AM, *n* = 6). Collectively, these results suggest that exogenous or endogenous activation of CB_1_Rs in the upper small intestinal epithelium inhibits nutrient-induced CCK secretion.

**Table 1:** Impact of diet on monoacylglycerols in mouse small-intestinal epithelium.

MAG	20:4 (2-AG) (nmol g^−1^)	18:1 (nmol g^−1^)	18:2 (nmol g^−1^)	16:0 (nmol g^−1^)	22:6 (nmol g^−1^)	Total (nmol g^−1^)
SD	80.23 ± 8.542	49.57 ± 9.804	217.7 ± 52.09	33.99 ± 4.125	9.413 ± 1.996	390.9 ± 72.24
WD	132.5 ± 22.20	109.0 ± 22.03	415.3 ± 83.26	150.1 ± 21.53	22.63 ± 3.703	829.4 ± 144.2
*p*	**0.0353**	**0.0206**	0.0554	**<0.0001**	**0.0049**	**0.0122**

MAG, Monoacylglycerol represented as fatty acid chain; SD, Standard diet *n* = 10; WD, Western diet *n* = 9. Mean values are shown as ± S.E.M. Bold values are significantly different determined by two-tailed unpaired *t*-test.

All levels of CCK-8 in these experiments fell within the range of the standard curve for CCK-8 quantitation by a sensitive and selective CCK-8 ELISA, which shows no cross-reactivity for gastrin (see Supplementary Figure S3), another gut-derived peptide that shares some common molecular features with CCK-8 (Walsh et al., 1982; Eysselein et al., 1984; Wolfe et al., 1985; Shulkes and Baldwin, 1997). Furthermore, the range of CCK-8 levels in our studies (from 0.27 ± 0.02 to 0.8 ± 0.03 ng per ml or 0.23 ± 0.02 to 0.7 ± 0.03 nM) aligns with reported *K_i_* and EC_50_ values of sulfated CCK-8 in several binding and *in vitro* bioassays (e.g., amylase release from pancreatic acini and ileum contractions in guinea pig) (Charpentier et al., 1988).

CB_1_R activation is reported to decrease gastric emptying, an effect also found in mice fed a high-fat diet for 14 weeks (Pertwee, 2001; Di Marzo et al., 2008). To identify if altered gastric emptying occurs under our conditions and may contribute in part to inhibited corn oil-induced CCK release, we developed a novel UPLC/MS/MS-based method to evaluate if CB_1_R activation with WIN 55,212-2 or exposure to WD for 60 days impacts gastric emptying following oral gavage of corn oil in SD and WD mice, respectively. Thirty minutes after administration of drugs, we administered by oral gavage corn oil (500 μl) that contained 19:2 free-fatty acid (1 nmol) as a tracer and measured by UPLC/MS/MS the remaining quantities of 19:2 free-fatty acid in the stomach 30 min after gavage. WIN 55,212-2 (3 mg per kg) alone or in combination with AM6545 (10 mg per kg) had no effect on gastric emptying of corn oil in SD mice (see Supplementary Figure S4A). Similarly, WD mice displayed no changes in gastric emptying of corn oil when compared to SD mice (see Supplementary Figure S4B). These data suggest that exogenous activation (WIN in SD mice) or endogenous activation (elevated small intestinal epithelial eCB levels in WD mice) of CB_1_Rs does not affect gastric emptying of corn oil under our conditions, and does not likely impact CCK release by a mechanism that includes alterations in gastric emptying in mice.

CB_1_Rs in pancreatic beta cells control insulin release and glucose homeostasis (Juan-Pico et al., 2006; De Petrocellis et al., 2007; Bermudez-Silva et al., 2008; Nakata and Yada, 2008; Li et al., 2010; Gonzalez-Mariscal et al., 2018). Thus, we tested if drug treatment impacted glucose levels in response to corn oil gavage in SD mice, which in turn, could affect gastric emptying, motility, or enteroendocrine hormones from small intestinal enteroendocrine cells. Glucose levels in blood were collected from tail vein and monitored *via* hand-held glucose monitor at (1) time of drug administration, (2) 30 min later just prior to corn oil gavage, and (3) 30 min later at time of kill (See Supplementary Figure S5). Drug treatment had no significant impact on blood glucose levels at any time point prior to or after gavage of corn oil (See Supplementary Figure S5). These data suggest that, under our conditions, activating CB_1_Rs does not impact blood glucose levels following oral gavage of corn oil in mice.

### Activity of Enzymes Responsible for Metabolism of 2-AG and Other Monoacylglycerols Is Dysregulated in the Upper Small-Intestinal Epithelium in Mice Chronically Fed WD

**Figure 4:**
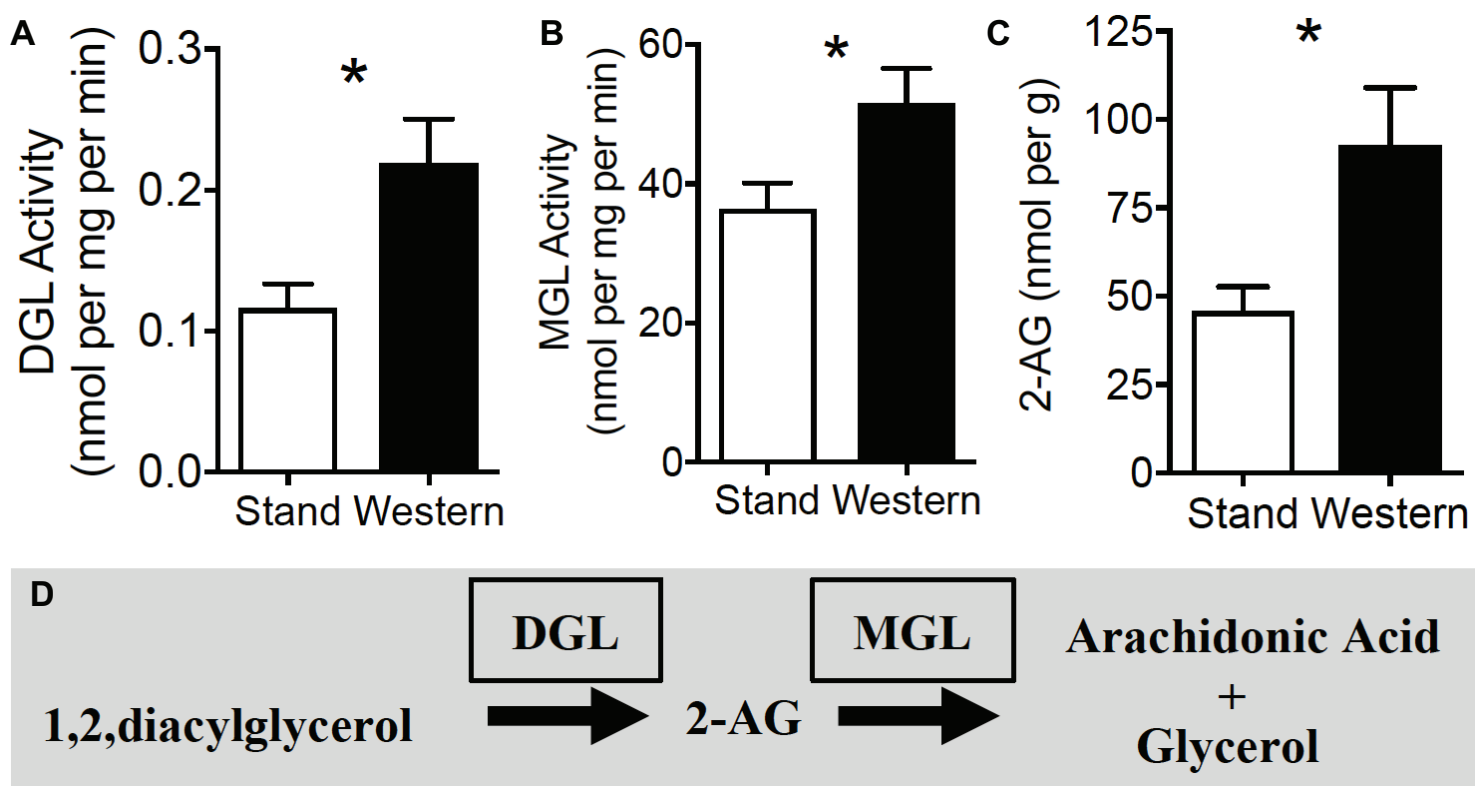
2-AG biosynthesis and degradation are upregulated in small intestine during obesity. Hydrolytic activity of DGL **(A)** and MGL **(B)** are increased in mice maintained on western diet (WD) when compared to controls fed a standard diet (SD). Levels of the endocannabinoid, 2-AG, are increased in jejunum mucosa of WD mice, when compared to SD mice **(C)**. 2-AG is formed by the hydrolysis of its 1,stearoyl,2-arachidonoyl-*sn*-glycerol precursor by DGL and is subsequently degraded by MGL into arachidonic acid and glycerol **(D)**. Data expressed as mean ± S.E.M. Analyzed using Student’s two-tailed *t*-test. *n* = 6 per condition, *p < 0.05.

We next aimed to identify the mechanism(s) of increased 2-AG and related monoacylglycerol levels (see Table 1) in WD mice by analyzing activity of their biosynthetic [diacylglycerol lipase (DGL)] and degradative enzymes [monoacylglycerol lipase (MGL)] using our lab’s UPLC/MS/MS-based functional enzyme assay methods (see Supplementary Figure S6 for validation of enzyme assays). When compared to SD mice, WD mice displayed an increase in activity of DGL (Figure 4A; SD = 0.12 ± 0.02, WD = 0.22 ± 0.03 nmol per mg protein per min; *p* = 0.016, reactions from six mice per diet group), and MGL (Figure 4B; SD = 36.32 ± 3.82, WD = 51.60 ± 4.95 nmol per mg protein per min; *p* = 0.035, reactions from six mice per diet group) in isolated tissue from the upper small intestinal epithelium. Congruent with data in Table 1 and (Argueta and DiPatrizio, 2017), these effects were met with increased levels of 2-AG in upper small intestinal epithelium of separate mice (Figure 4C; SD = 45.71 ± 6.93, WD = 92.57 ± 16.41 nmol per g; *p* = 0.014, *n* = 9–10 per diet group). See Figure 4D for diagram of the 2-AG metabolic pathways. Together, these results suggest that monoacylglycerol metabolic pathways are dysregulated after chronic exposure to WD, which leads to a net increase in monoacylglycerols, including 2-AG, in the upper small-intestinal epithelium.

### Expression of Select eCB System Components in the Upper Small-Intestinal Epithelium Is Dysregulated in Mice Chronically Fed WD and Partially Conserved in CCK-Positive Cells

**Figure 5:**
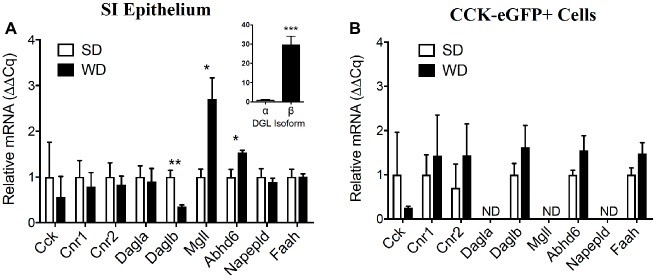
Expression of select components of the eCB system is dysregulated in the upper small intestine of DIO mice and partially conserved in CCK-eGFP+ cells. Expression of mRNA for cholecystokinin (Cck), cannabinoid receptor subtype 1 (Cnr1) and 2 (Cnr2), and other components of the eCB system in upper small-intestinal mucosal scrapings are not impacted by western diet (WD) exposure when compared to controls fed a standard diet (SD) **(A)**. Expression of diacylglycerol lipase beta (Daglb) is decreased, and expression of the degradative enzymes monoacylglycerol lipase (Mgll) and alpha-beta hydrolyzing domain 6 (Abhd6) are increased in WD mice. Expression of mRNA for CCK or components of the eCB system were not significantly affected by diet in eGFP (+) sorted cells **(B)**. Expression of mRNA for diacylglycerol lipase alpha (Dagla), Mgll, and N-acyl phosphatidylethanolamine specific phospholipase D (Napepld) was not detected (ND) **(B)**. Data expressed as mean ± S.E.M. Analyzed using Student’s two-tailed *t*-test. *n* = 3 per group in triplicate and *p < 0.05, **p < 0.01, ***p < 0.001 **(A)**; *n* = 3 per group in triplicate, *p* > 0.05 **(B)**.

Relative expression of mRNA for intestinal CCK, CB_1_Rs, and CB_2_Rs (Cnr2) was unchanged in whole upper small intestinal epithelial scrapings from mice fed WD versus SD mice (Figure 5A; CCK, SD = 1.00 ± 0.76, WD = 0.56 ± 0.45, *p* = 0.64; Cnr1, SD = 1.00 ± 0.36, WD = 0.79 ± 0.31, *p* = 0.67; Cnr2, SD = 1.00 ± 0.31, WD = 0.83 ± 0.19, *p* = 0.65; data from four mice per diet group). Expression of mRNA for the alpha isoform of DGL (Dagla) was also unaffected by diet (Figure 5A; SD = 1.00 ± 0.25, WD = 0.90 ± 0.29, *p* = 0.80); however, expression of mRNA for the beta isoform of DGL (Daglb) was reduced in WD versus SD mice (Figure 5A; SD = 1.00 ± 0.15, WD = 0.35 ± 0.03, *p* = 0.005), while mRNA for MGL (Mgll) and the serine hydrolase alpha/beta hydrolase domain 6 (Abhd6) were increased in small intestinal epithelium under the same conditions (Figure 5A; Mgll, SD = 1.00 ± 0.17, WD = 2.71 ± 0.46, *p* = 0.013; Abhd6, SD = 1.00 ± 0.16, WD = 1.54 ± 0.05, *p* = 0.02). No changes were found for the fatty acid ethanolamide biosynthetic enzyme, NAPE-PLD, or the fatty acid ethanolamide degradative enzyme, FAAH (Figure 5A; NAPE-PLD, SD = 1.00 ± 0.18, WD = 0.89 ± 0.08, *p* = 0.6; FAAH, SD = 1.00 ± 0.17, WD = 1.00 ± 0.07, *p* = 0.99). Furthermore, the upper small-intestinal epithelium is enriched in expression of mRNA for Daglb when compared to Dagla (Figure 5A inset; Dagla = 1.00 ± 0.19, Daglb = 29.73 ± 4.3; *p* = 0.001; data from four mice fed SD).

It is important to note, in contrast to our previous report that included analysis of eCB system expression in the upper small intestinal epithelium of mice maintained on WD and SD [Lab Diet 5001 used in (Argueta and DiPatrizio, 2017)], in this study we used a soy protein-free Teklad 2020x as a control SD in order to eliminate any potential effects of phytoestrogen-containing soy protein on eCB metabolism or behavior [see (McFarland et al., 2004; Thors et al., 2007, 2008; Peroni et al., 2012)]. We found two differences in results when comparing use of the two control diets versus WD. We reported no changes in expression of mRNA for the beta isoform of DGL and increases in expression of mRNA for FAAH in WD mice when compared to control SD mice (Argueta and DiPatrizio, 2017); however, in this study, we found decreased expression of mRNA for the beta isoform of DGL and no changes in expression of mRNA for FAAH in WD mice when compared to SD mice. These differences highlight possible effects of diets that utilize soy protein on expression of eCB metabolic enzymes and eCB metabolic function. A direct comparison of the impact of specific control diets on expression of eCB system components, however, remains to be evaluated.

CCK-eGFP-positive cells isolated from mice fed SD or WD mice displayed no differences between diet condition in expression of mRNA for CCK and components of the eCB system that include Cnr1, Cnr2, Daglb, Abhd6, and FAAH (Figure 5B; *p* > 0.05 not significant, data from three mice per diet group). Dagla, Mgll, and Napepld mRNA were below detectable levels, which suggest a lack of expression of these eCB system components in CCK-containing cells.

Collectively, these results identify select eCB system gene transcripts in CCK-containing cells, and changes in expression of biosynthetic and degradative enzyme gene transcripts in whole epithelium of WD mice that do not fully correspond to changes in activity of their proteins, including DGL and MGL (see Figure 4). The latter suggests possible post-transcriptional and/or post-translational changes in expression of these enzymes in the upper small-intestinal epithelium in WD mice when compared to lean SD mice, although this hypothesis remains to be directly tested. Furthermore, a lack of expression of the fatty acid ethanolamide (FAE) biosynthetic enzyme, NAPE-PLD, in CCK-containing cells suggests that FAEs including anandamide—which is also found in small intestinal epithelium of rodents (Izzo et al., 2009; DiPatrizio et al., 2011, 2013, 2015; Argueta and DiPatrizio, 2017; Perez and DiPatrizio, 2018)—is generated in neighboring cells and therefore may act in a paracrine manner with I-cells that contain CB_1_Rs. In contrast, expression of mRNA for the beta isoform of the monoacylglycerol biosynthetic enzyme, DGL, is abundantly expressed in CCK-containing cells, which suggests that 2-AG may signal at CB_1_Rs in an autocrine manner at these cells. Expression of the primary 2-AG degradative enzyme, MGL, is absent in CCK-containing I-cells, which suggests that 2-AG is degraded at adjacent cells and therefore may additionally signal CB_1_Rs on adjacent cells in a paracrine manner. A comprehensive analysis of eCB system architecture and its cell-specific expression in the upper small-intestinal epithelium of mice remains for future studies.

### Western Diet Exposure for 60 Days Is Associated With Obesity and Hyperphagia in Male Mice

**Figure 6:**
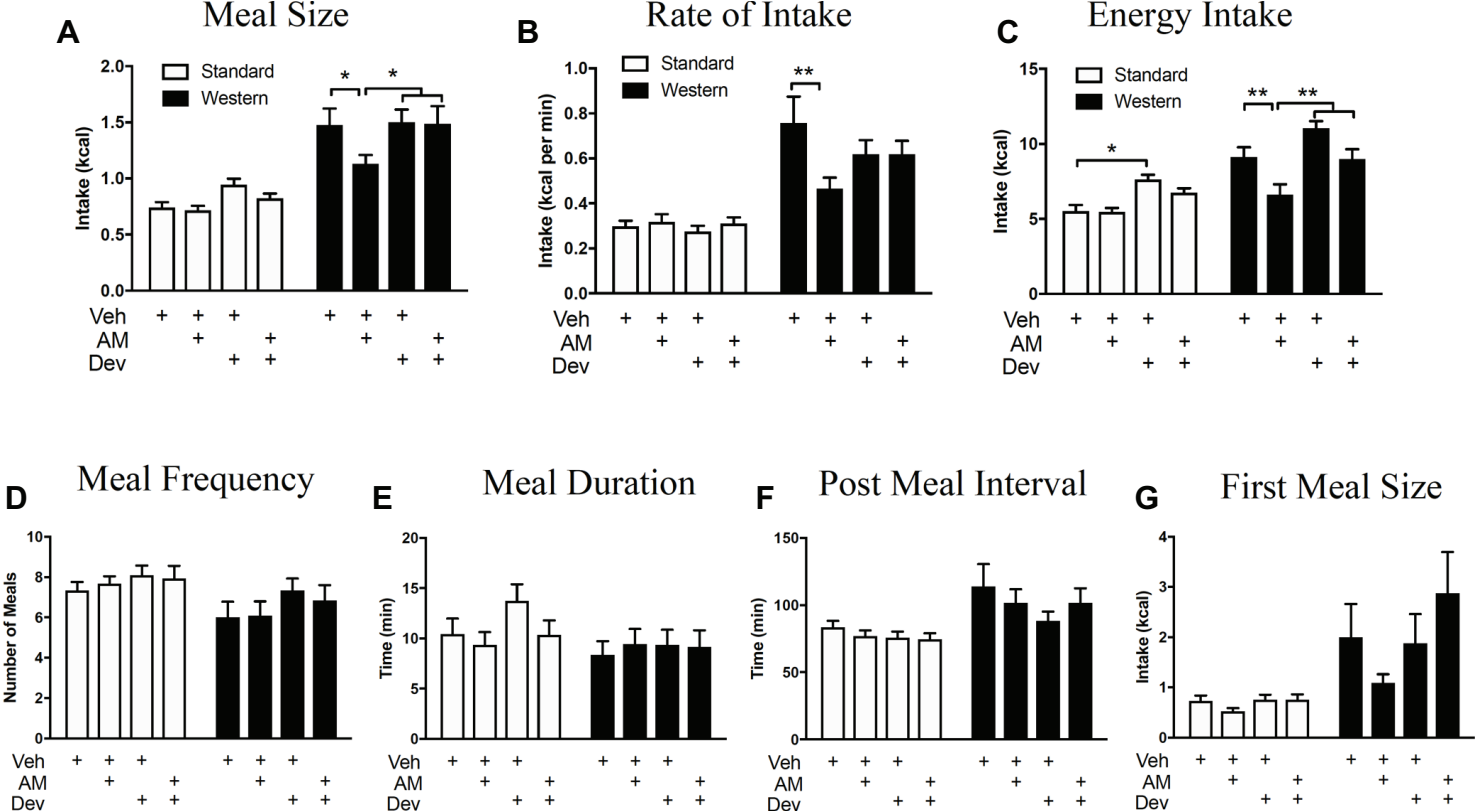
Peripheral eCB signaling drives hyperphagia in mice maintained on a WD *via* a CCK-dependent mechanism. Caloric intake **(A)**, meal size **(B)**, and rate of intake **(C)** of a western diet (closed bars) are significantly reduced during a 12 h test following inhibition of peripheral CB_1_Rs with AM6545 (AM, 10 mg per kg), an effect absent in low-fat chow fed mice (open bars) and that is blocked by co-administration with the CCK_A_ receptor antagonist, devazepide (Dev; 0.1 mg per kg). Diet and drug had no effect on meal frequency **(D)**, meal duration **(E)**, post meal interval **(F)**, or first meal size **(G)**. All data represented as means ± SEM. Analyzed using regular 2-Way ANOVA with *post hoc* Newman-Keuls multiple comparison’s test. *n* = 11–12 per condition, *p < 0.05, **p < 0.01.

Consistent with our previous studies (Argueta and DiPatrizio, 2017), exposure to WD for 60 days, when compared to lean mice fed SD for 60 days, was associated with (1) a rapid and sustained increase in body mass when compared to control mice fed SD for 60 days, (2) increased 24 h meal size, (3) rate of food intake, and (4) total 24 h caloric intake (see Supplementary Figure S7 and Table 2 for details and data). No significant changes were found for other feeding behaviors including (1) first meal size, (2) meal frequency, (3) meal duration, and (4) post-meal interval. As discussed above in “Expression of Select eCB System Components in the Upper Small Intestinal Epithelium is Dysregulated in Mice Chronically Fed WD and Partially Conserved in CCK-Positive Cells,” in contrast to our previous study (Argueta and DiPatrizio, 2017), in this study we used a soy-protein-free lab chow. Irrespective of control diet, however, WD intake was consistently associated with increased 2-AG levels (Table 1) and hyperphagia across relevant parameters in both studies (Table 2, Figure 6; Argueta and DiPatrizio, 2017). Together, these data suggest that exposure to a WD rapidly induces body weight gain that is met with increased meal size, rate of intake, and daily caloric intake, when compared to lean controls.

**Table 2:** Consumption of WD is associated with hyperphagia.

	Δ Body mass (g)	Meal size (kcal)	Intake rate (kcal min^−1^)	24 h Intake (kcal)	First meal (kcal)	Frequency (meals day^−1^)	Duration (Min)	PMI (Min)
SD	8.90 ± 0.31	0.69 ± 0.04	0.33 ± 0.02	8.10 ± 0.61	0.72 ± 0.11	11.67 ± 0.45	8.61 ± 1.16	114.7 ± 8.4
WD	18.14 ± 0.46	1.29 ± 0.10	0.71 ± 0.08	13.28 ± 0.81	1.99 ± 0.66	9.83 ± 1.42	6.85 ± 0.98	129.3 ± 14.3
*p*	**<0.0001**	**<0.0001**	**<0.0001**	**<0.0001**	0.07	0.23	0.26	0.31

*PMI, Post meal interval. Mean values are shown as ± S.E.M. *n* = 10. Bold values are significantly different determined by two-tailed unpaired *t*-test*.

### Pharmacological Inhibition of CCK_A_ Receptors Blocks the Anorexic Effect of AM6545 in Mice Chronically Fed a WD

We next tested the hypothesis that peripheral CB_1_Rs control feeding behavior by a mechanism that includes control of CCK-mediated satiation signaling. When compared to vehicle treatment in mice fed WD for 60 days, AM6545 treatment (10 mg per kg) in WD mice reduced meal size (Figure 6A; vehicle = 1.47 ± 0.15 kcal, AM6545 = 1.13 ± 0.67 kcal; *p* < 0.05, *n* = 12), rate of intake (Figure 6B; vehicle = 0.76 ± 0.12 kcal per min, AM6545 = 0.46 ± 0.05 kcal per min; *p <* 0.01), and total caloric intake (Figure 6C; vehicle = 9.11 ± 0.67 kcal per min, AM6545 = 6.62 ± 0.69 kcal per min; *p* < 0.01) during a 12 h test, which is consistent with our previous findings (Argueta and DiPatrizio, 2017). Furthermore, AM6545 treatment in mice fed SD for 60 days did not affect meal size (Figure 6A; vehicle = 0.74 ± 0.05 kcal, AM6545 = 0.71 ± 0.04 kcal; *p* > 0.05, *n* = 12), rate of intake (Figure 6B; vehicle = 0.30 ± 0.03 kcal per min, AM6545 = 0.32 ± 0.03 kcal per min; *p* > 0.05), and total caloric intake (Figure 6C; vehicle = 5.51 ± 0.42 kcal per min, AM6545 = 5.45 ± 0.28 kcal per min; *p* > 0.05) during a 12 h test. Importantly, co-administration of a low dose of the CCK_A_ receptor antagonist, devazepide (Dev; 0.1 mg per kg), in WD mice blocked the effects of AM6545 on reducing meal size (Figure 6A; vehicle = 1.47 ± 0.15 kcal, AM6545 + devazepide = 1.49 ± 0.16 kcal; *p* > 0.05), rate of intake (Figure 6B; vehicle = 0.76 ± 0.12 kcal per min, AM6545 + devazepide = 0.62 ± 0.06 kcal per min; *p* > 0.05), and total caloric intake (Figure 6C; vehicle = 9.11 ± 0.67 kcal per min, AM6545 + devazepide = 8.98 ± 0.67 kcal per min; *p* > 0.05). Administration of devazepide alone affected only total 12-h caloric intake in SD mice (Figure 6C; vehicle = 5.51 ± 0.42 kcal per min, devazepide = 7.61 ± 0.33 kcal per min; *p <* 0.05). Neither AM6545 nor devazepide affected other meal parameters including meal frequency (Figure 6D), meal duration, (Figure 6E), post-meal interval (Figure 6F), or first-meal size (Figure 6G) in SD or WD mice. These data suggest that the acute anorexic effects of AM6545 in WD mice are dependent on a mechanism that includes activation of CCK_A_ receptors and inhibition of gut-brain satiation signaling.

## Discussion

**Figure 7:**
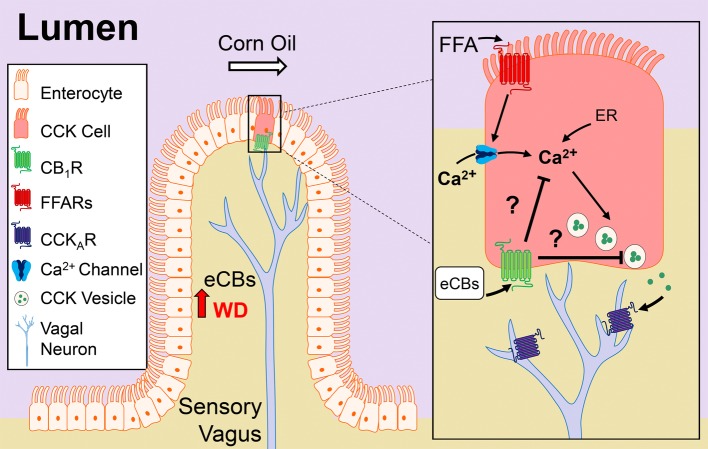
Model of CB1R control of nutrient-induced CCK release. Our studies suggest that cannabinoid CB_1_Rs in the upper small-intestinal epithelium control nutrient-induced satiation signaling, and their signaling is increased in diet-induced obesity, which drives overeating by delaying satiation. The upper small-intestinal epithelium contains enteroendocrine I-cells, which are a subpopulation of enterocytes that secrete cholecystokinin (CCK) when nutrients – including dietary fats – enter the lumen (Rehfeld, 1998; Tanaka et al., 2008; Wang et al., 2011; Liou et al., 2011a; Brennan et al., 2012). Dietary fats (e.g., corn oil), in the form of triacylglycerols, are hydrolyzed in the lumen into mostly monoacylglycerols and free-fatty acids (FFAs) that are sensed by free-fatty acid receptors (FFARs) located on enteroendocrine cells in the small-intestinal epithelium. Activation of FFARs stimulates secretion of CCK by a mechanism that requires calcium (Ca^2+^) influx and/or intracellular (i.e., endoplasmic reticulum, ER) mobilization (McLaughlin et al., 1998; Hira et al., 2004; Tanaka et al., 2008). CCK activates CCK_A_ receptors located on adjacent afferent sensory vagal fibers, which in turn, communicate with the brain and control meal size and satiation (Smith et al., 1981, 1985; Schwartz and Moran, 1994; Kaelberer et al., 2018). Consumption of a Western diet (WD) is associated with increased levels of the endocannabinoids (eCBs) and their activity at CB_1_Rs in the upper small-intestinal epithelium, which we propose inhibits CCK release and satiation signaling. The molecular mechanism(s) mediating CB_1_R control of CCK release is unknown, but may include inhibition of Ca^2+^-mediated CCK release. A future test of this hypothesis is warranted.

The molecular underpinnings of gut-brain signaling and their dysregulation in DIO are poorly defined. Our studies suggest that eCB activity at CB_1_Rs in the upper small intestinal epithelium is upregulated in mice chronically fed a WD, which in turn, promotes overeating by a mechanism that includes inhibiting nutrient-induced gut-brain satiation signaling (see Figure 7 for model). Six primary findings support this conclusion: (1) CB_1_Rs are enriched in CCK-containing cells in the mouse upper small intestinal epithelium; (2) oral gavage of corn oil increased circulating levels of CCK-8 in lean mice, and pharmacological activation of CB_1_Rs blocked this effect, which was reversed by inhibition of peripheral CB_1_Rs with a peripherally-restricted CB_1_R neutral antagonist; (3) levels of 2-AG and other monoacylglycerols were increased in the upper-small intestinal epithelium of WD mice when compared to lean mice, and this effect was associated with dysregulated monoacylglycerol metabolism; (4) oral gavage of corn oil failed to affect circulating levels of CCK-8 in WD mice, and inhibition of peripheral CB_1_Rs in WD mice restored the ability for corn oil to increase CCK levels; (5) pharmacological inhibition of peripheral CB_1_Rs in WD mice blocked overeating associated with increased meal size, rate of feeding, and total caloric intake; and (6) the hypophagic effects of peripheral CB_1_R antagonism in WD mice were reversed by pretreatment with a low-dose CCK_A_ receptor antagonist. Collectively, our studies identify a previously unknown role for the eCB system at the interface of nutrient-sensing and gut-brain satiation signaling that becomes dysregulated in DIO and promotes overeating by delaying satiation.

Our studies suggest that the eCB system in the small intestinal epithelium controls feeding behavior by a mechanism that includes inhibiting nutrient-induced release of the gut-derived satiation peptide, CCK, which in turn increases meal size and caloric intake. CCK is secreted from enteroendocrine I-cells in the upper small intestinal epithelium after nutrients arrive in the lumen (McLaughlin et al., 1999; Raybould et al., 2006; Steinert et al., 2017; Kaelberer et al., 2018) and controls meal size and induces satiation by activating CCK_A_ receptors on afferent vagal fibers (Smith et al., 1981, 1985; Schwartz and Moran, 1994; Raybould, 2007; Kaelberer et al., 2018; Schwartz, 2018) and possibly the brain (Reidelberger et al., 2004; Ripken et al., 2015). Indeed, polymorphisms in CCK_A_ receptor genes in humans are associated with increased meal size and food intake, and obesity (Miller et al., 1995; Marchal-Victorion et al., 2002; de Krom et al., 2007). Furthermore, CCK in a stabilized form resistant to degradation in the GI tract is effective at reducing food intake and body weight in DIO rodents (Pierson et al., 1997; Irwin et al., 2012, 2013b), and activating CCK_A_ receptors enhances the anti-obesity properties of GLP-1 agonists, amylin, and leptin (Trevaskis et al., 2010, 2015; Irwin et al., 2013a, 2015).

Gene transcripts and immunoreactivity for CB_1_Rs were found in CCK-containing I-cells in the upper small-intestinal epithelium of mice (see Figures 1, 2; Sykaras et al., 2012). Furthermore, the hypophagic effects of AM6545 were blocked by co-administration of the CCK_A_ receptor antagonist, devazepide. These results suggest that when eCB activity is elevated at local CB_1_Rs in the upper small-intestinal epithelium in DIO, increased CB_1_R activation may inhibit nutrient-induced release of satiation peptides from small-intestinal enteroendocrine cells and lead to increased meal size and caloric intake. In support of this hypothesis, oral gavage of corn oil—which potently increases circulating levels of bioactive CCK-8 in lean mice that have low levels of small-intestinal eCB levels—failed to affect circulating levels of CCK-8 in mice chronically fed WD that have elevated eCB levels in the small-intestinal epithelium. Moreover, inhibiting elevated eCB signaling at peripheral CB_1_Rs with AM6545 in WD mice—at a dose that blocked overeating—restored the ability for corn oil to increase circulating levels of CCK-8.

The mechanisms of CB_1_R control of nutrient-induced release of CCK from enteroendocrine I-cells in the upper small-intestinal epithelium are unknown. Nonetheless, a primary mechanism by which CB_1_Rs block neurotransmitter release is by inhibiting calcium influx or mobilization (Howlett et al., 2010; Pertwee, 2015), and nutrient-induced CCK release is calcium-dependent (McLaughlin et al., 1998; Gevrey et al., 2002; Hira et al., 2004; Liou et al., 2011b; Nakajima et al., 2012). Thus, CB_1_R activity may inhibit release of gut peptides by a mechanism that includes inhibiting calcium influx or mobilization; however, a direct test of this hypothesis remains to be performed (see Figure 7 for proposed mechanism).

It is controversial if obesity impacts CCK secretion [see for review, (Steinert et al., 2017)]. In line with our present findings in mice, several studies suggest that CCK secretion is reduced in obese humans: fasting CCK levels were lower than non-obese (Baranowska et al., 2000) and a trend toward lower CCK release after intra-duodenal infusions of oleic acid in overweight or obese subjects (Stewart et al., 2011). Fat-induced CCK secretion and satiation induced by CCK administration were also reduced in rats fed a high-fat diet (Duca et al., 2013). Other studies, however, reported no differences in CCK levels between obese or lean humans following a meal (Brennan et al., 2012), and increases in CCK after a high-fat meal (French et al., 1993). Furthermore, several preclinical studies in rodents suggest that sensitivity of vagal afferent neurons to the satiating effects of CCK may be decreased in DIO (Covasa et al., 2000; Daly et al., 2011; Duca et al., 2013; de Lartigue, 2016). This phenomenon may be due, in part, to changes in membrane properties of neurons in the nodose ganglion. The satiating actions of a physiological dose of CCK, however, were equally effective in suppressing food intake in obese and lean human subjects (Lieverse et al., 1995). Moreover, a variety of studies conducted over the past several decades show that CCK-induced satiation is mediated by the vagus nerve (Smith et al., 1981, 1985; Schwartz and Moran, 1994; Raybould, 2007; Dockray, 2013; Kaelberer et al., 2018; Schwartz, 2018); however, selected studies show that gut-derived CCK may additionally interact with CCK-A receptors in the brain (Reidelberger et al., 2004; Ripken et al., 2015). We used the brain-penetrant CCK-A receptor antagonist, devazepide, in our studies; therefore, we cannot rule out the possibility that CCK-A receptors in the brain participate in the appetite-suppressing effects of CCK release following inhibition of peripheral CB_1_Rs. Thus, given discrepancies in the literature regarding the underlying mechanisms of gut-brain signaling and its dysregulation in DIO, it is critical to examine the impact of diet and obesity on gut-brain satiation signaling using reliable and reproducible model systems.

It is plausible that CB_1_R control of nutrient-induced CCK release is one of several mechanisms by which peripheral CB_1_Rs impact gut-brain signaling pathways (Burdyga et al., 2004, 2006; Cluny et al., 2013). For example, administration of ghrelin – which is produced in the stomach and upper small intestinal epithelium and increases feeding [see for review, (Steinert et al., 2017; Kaelberer and Bohorquez, 2018)] – blocked downregulation of CB_1_Rs in the nodose ganglion after, both, re-feeding and CCK administration in fasted rats (Burdyga et al., 2006). Moreover, pharmacological inhibition of CB_1_Rs blocked fasting-induced ghrelin production in rats (Cani et al., 2004; Al-Massadi et al., 2011; Senin et al., 2013), which suggests that CB_1_Rs in the upper GI tract may control ghrelin signaling. Furthermore, Kunos and colleagues reported that a peripherally-restricted CB_1_R inverse agonist improved a host of metabolic parameters as well as reducing food intake in DIO mice by a mechanism that may include reversing hyperleptinemia and leptin resistance associated with DIO (Tam et al., 2012) and restoring anorexic melanocortin signaling in the arcuate nucleus of the hypothalamus (Tam et al., 2017). Bellocchio and colleagues reported that the hypophagic effects of CB_1_R inhibition with the CB_1_R inverse agonist, rimonabant, is blocked by pharmacological inhibition of peripheral beta-adrenergic neurotransmission (Bellocchio et al., 2013), which suggests that CB_1_Rs may additionally control feeding behavior *via* interactions with the peripheral sympathetic nervous system. This study also showed that intact afferent vagal signaling was required for the hypophagic effects of rimonabant, and CB_1_Rs in the brain were not required for its pharmacological actions. Nonetheless, circulating levels of the eCBs increase in human and rodent models of obesity (Engeli et al., 2005; Bluher et al., 2006; Cote et al., 2007; Di Marzo et al., 2009; Matias et al., 2012; Argueta and DiPatrizio, 2017; Hillard, 2017; Simon and Cota, 2017; Little et al., 2018), which may directly interact with CB_1_Rs in the brain and control feeding behavior and energy homeostasis. A comprehensive analysis of this possibility remains to be performed. In addition to I-cells in the small intestinal epithelium (see Figures 1, 2; Sykaras et al., 2012), CB_1_Rs are also expressed in K-cells that produce and secrete glucose-dependent insulinotropic peptide [GIP (Moss et al., 2012; Reimann and Gribble, 2016)]. Pharmacological activation of CB_1_Rs inhibits GIP release in rodents, which suggests that local CB_1_Rs may impact glucose homeostasis *via* a mechanism that includes controlling nutrient-induced incretin release. Lastly, enteroendocrine cells in the intestinal lining form functional synapses with afferent vagal fibers (Kaelberer et al., 2018). Termed “neuropods” by Bohorquez and colleagues, these cells sense nutrients and release glutamate and CCK in a coordinated manner that interact with corresponding receptors on local afferent vagal fibers, which in turn, communicate with the brain. Our data suggest that CB_1_Rs may be at the interface of this signaling. It is unknown, however, if CB_1_Rs control glutamate signaling at these synapses in the small intestine as they do in the brain (Jung et al., 2007). Collectively, these studies—in combination with the present report—describe key roles for peripheral CB_1_Rs in feeding behavior and energy homeostasis.

In summary, our results provide evidence of a previously unknown mechanism of CB_1_R-mediated inhibition of gut-brain satiation signaling in DIO that promotes overeating. Pharmacological manipulation of these pathways in the periphery may provide a therapeutic advantage for the treatment of obesity and related metabolic disorders when compared to anti-obesity drugs that interact with the brain and display psychiatric side-effects (Christensen et al., 2007; Khera et al., 2016). Despite the peripherally-restricted properties of these CB_1_R antagonists, however, their impact on cognition and brain function by altering gut microbe activity is unknown and remains to be reported.

## Data Availability

The datasets generated for this study are available on request to the corresponding author.

## Ethics Statement

All procedures met the U.S. National Institute of Health guidelines for care and use of laboratory animals and were approved by the Institutional Animal Care and Use Committee of the University of California, Riverside.

## Author Contributions

DA and PP contributed to experimental design, performed experiments, collected data, processed data, and contributed to writing of the manuscript. AM provided the pharmacological compound, AM6545, and reviewed the manuscript. ND orchestrated the project, designed experiments, processed data, and wrote the manuscript.

### Conflict of Interest Statement

The authors declare that the research was conducted in the absence of any commercial or financial relationships that could be construed as a potential conflict of interest.

## Abbreviations

2-AG: 2-Arachidonoyl-*sn*-glycerol
Abhd6: alpha-beta-hydrolyzing domain 6
AEA: Anandamide
AM: AM6545
CB_1_R: Cannabinoid receptor subtype 1
CB_2_R: Cannabinoid receptor subtype 2
CCK: Cholecystokinin
CO: Corn oil
DAG: Diacylglycerol
Dev: Devazepide
DGL: Diacylglycerol lipase
DIO: Diet-induced obesity
eCB: Endocannabinoid
eGFP: Enhanced green fluorescent protein
FAAH: Fatty acid amide hydrolase
FACS: Fluorescence activated cell sorting
FAE: Fatty acid ethanolamide
MAG: Monoacylglycerol
MGL: Monoacylglycerol lipase
NAPE-PLD: N-acyl phosphatidylethanolamine-specific phospholipase D
SD: Standard diet
WD: Western diet
WIN: WIN 55,212-2.
