# Supplementary figures and images for: Cannabinoid CB1 Receptors Inhibit Gut-Brain Satiation Signaling in Diet-Induced Obesity
Source: Front Physiol. 2019 Jun 11;10:704. doi: 10.3389/fphys.2019.00704 (PMC6597959; doi:10.3389/fphys.2019.00704)

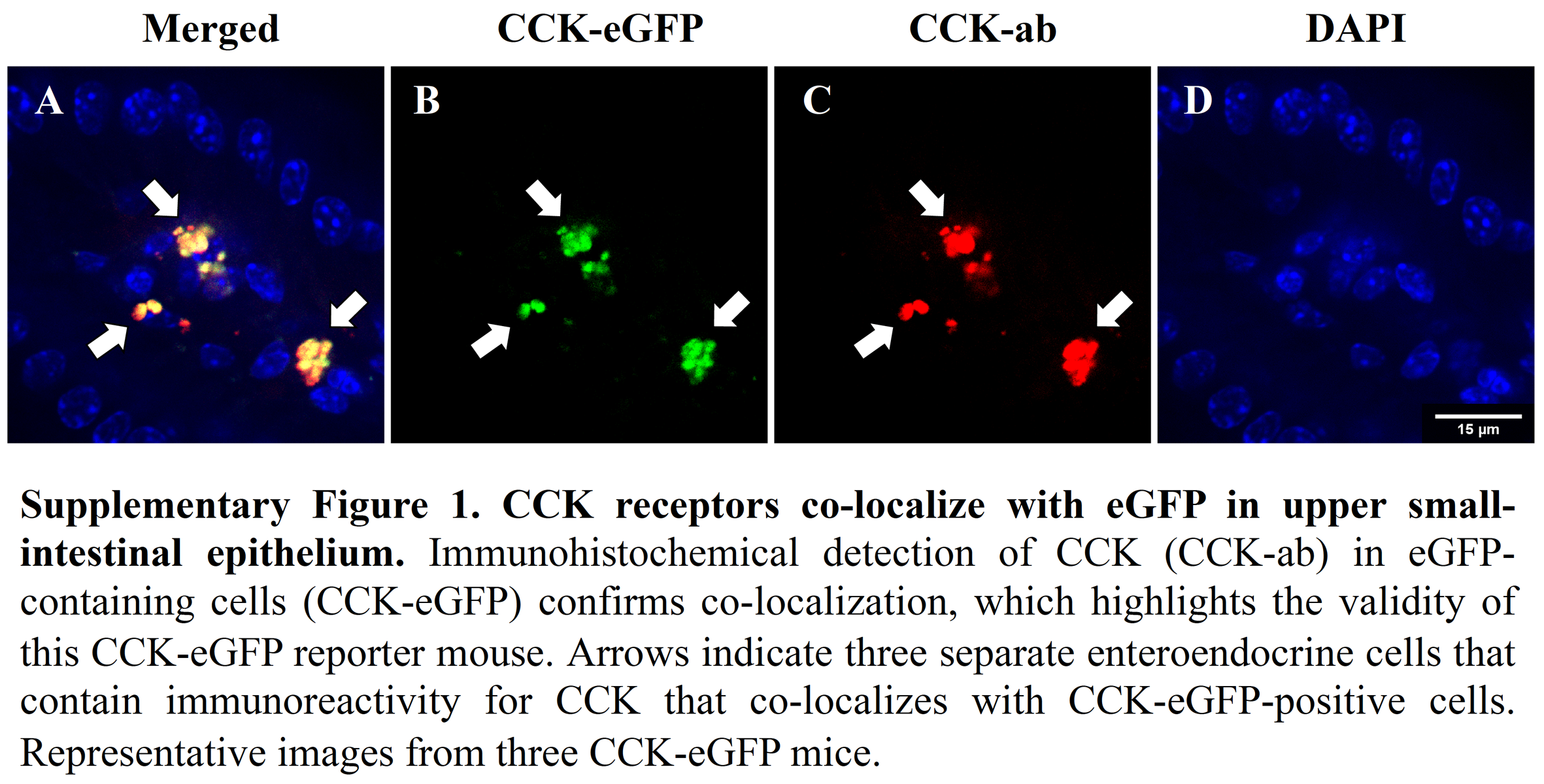

Supplement: Supplementary file 1 [file Image_1.TIF]

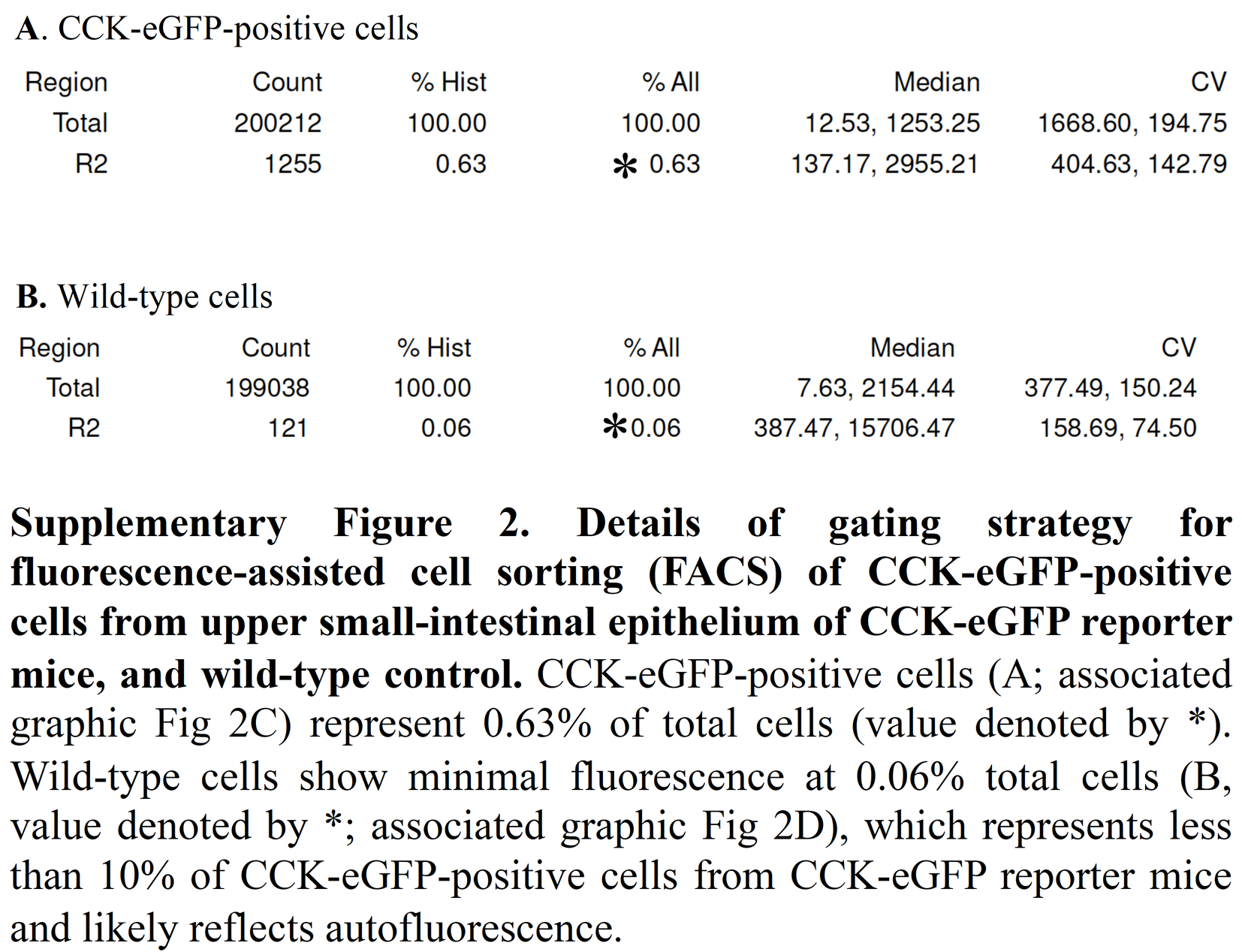

Supplement: Supplementary file 2 [file Image_2.tif]

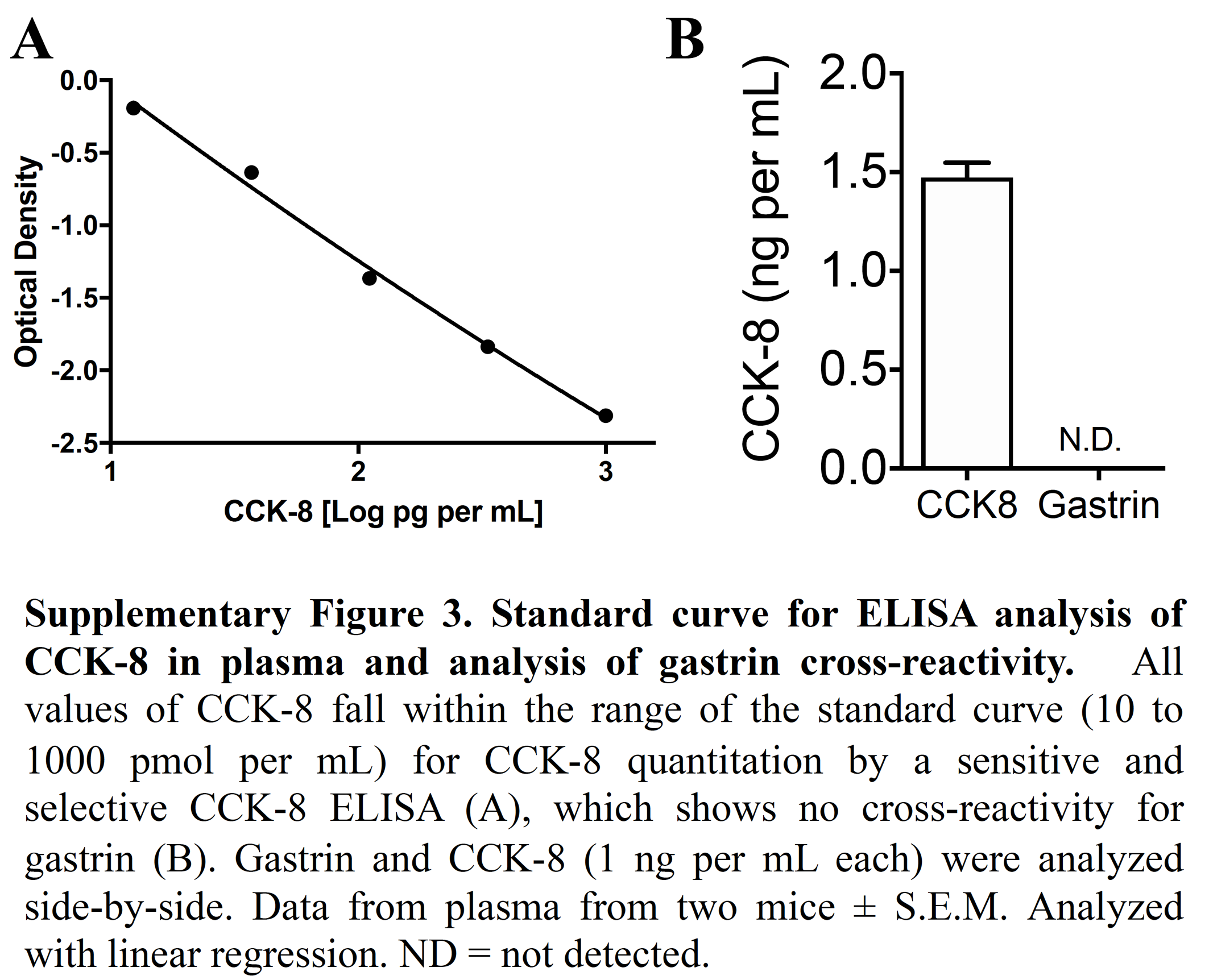

Supplement: Supplementary file 3 [file Image_3.TIF]

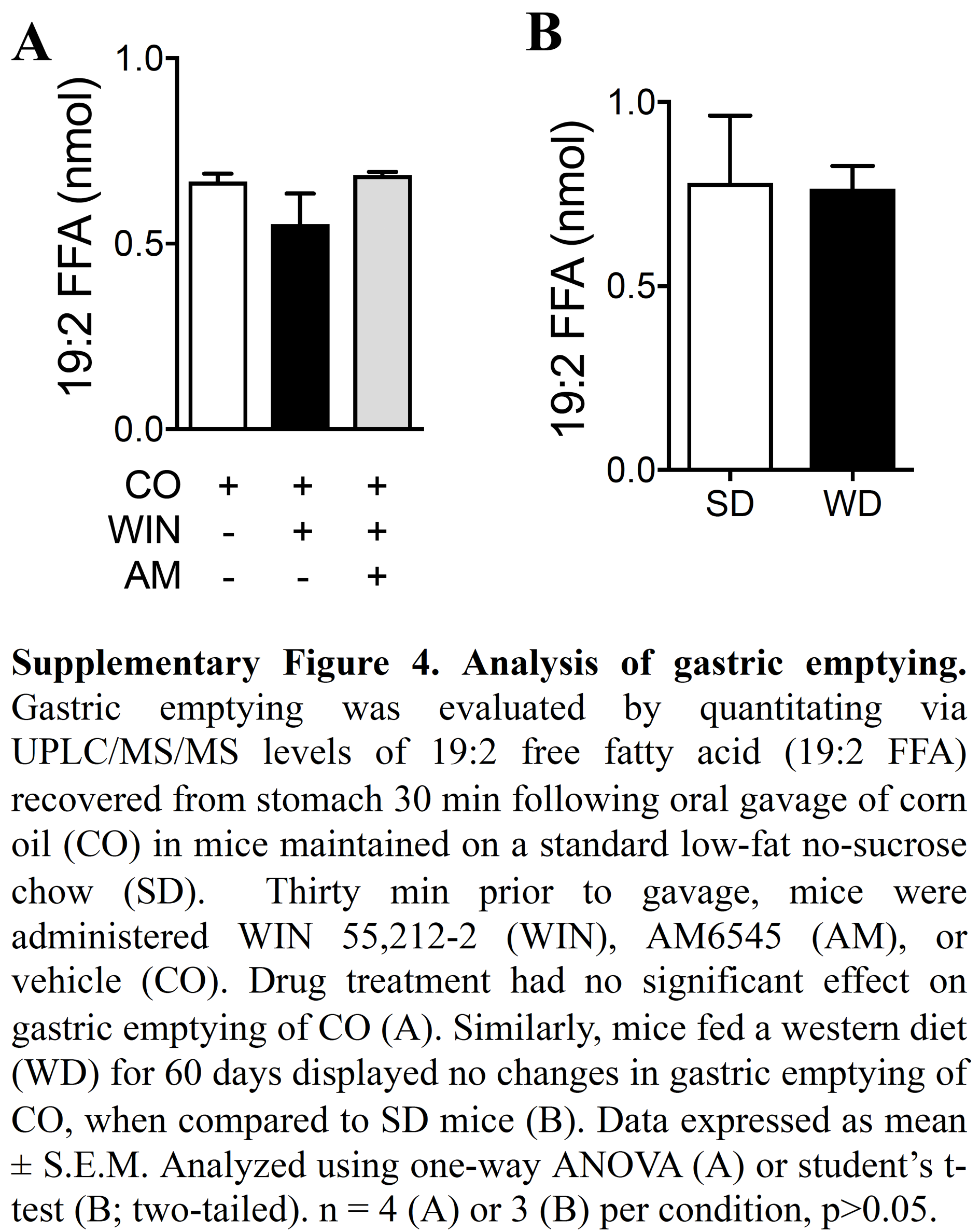

Supplement: Supplementary file 4 [file Image_4.TIF]

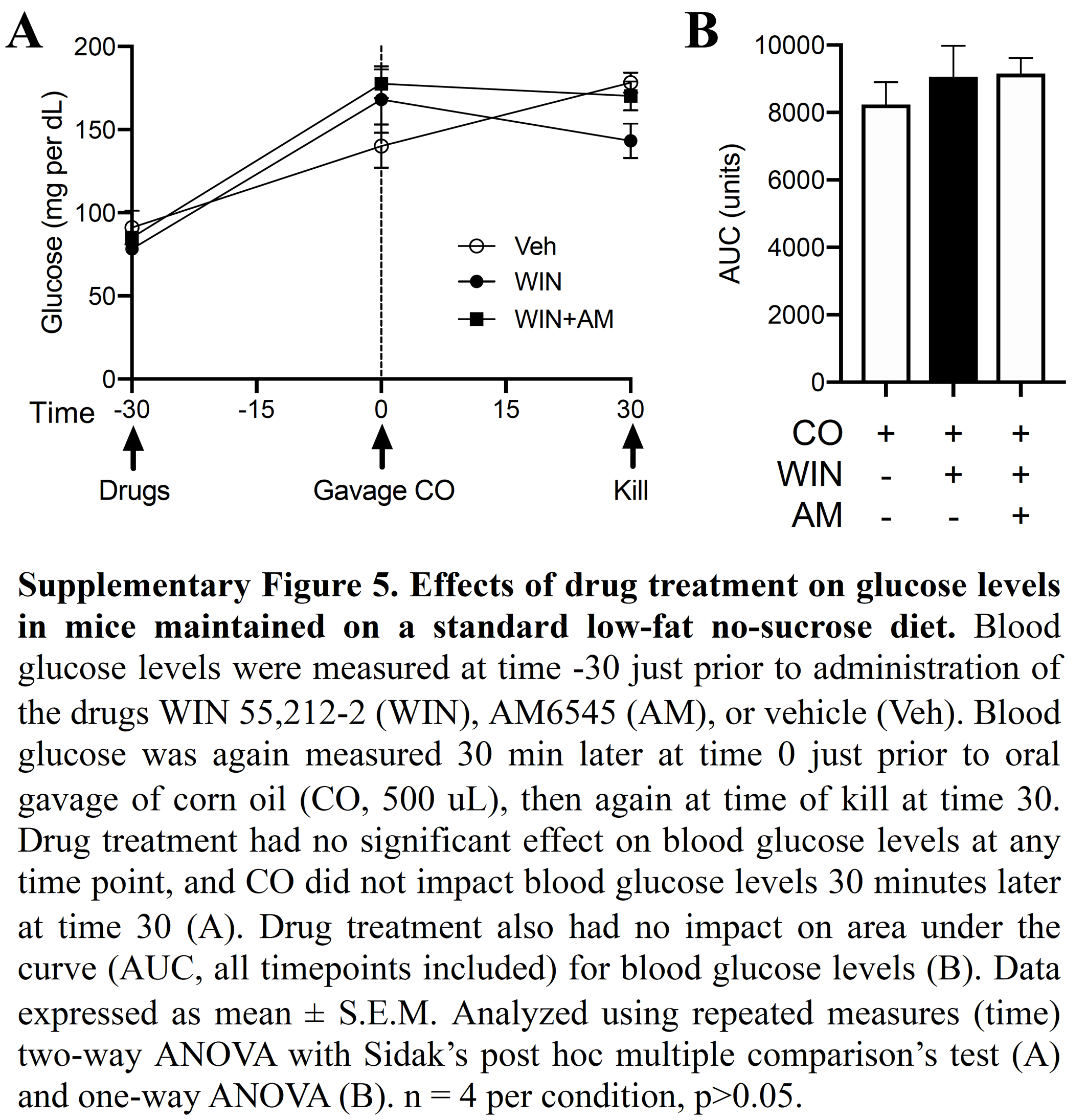

Supplement: Supplementary file 5 [file Image_5.TIF]

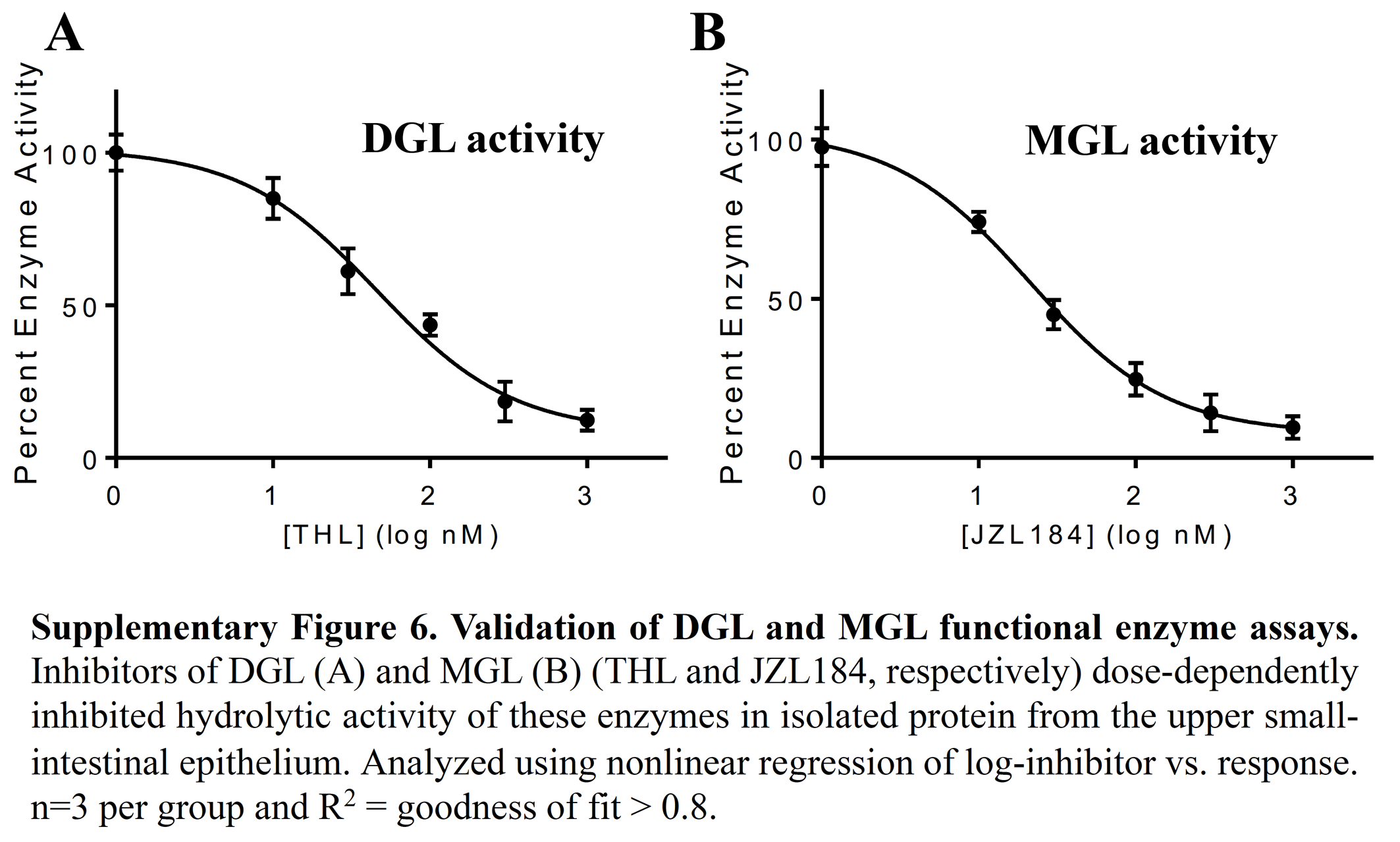

Supplement: Supplementary file 6 [file Image_6.TIF]

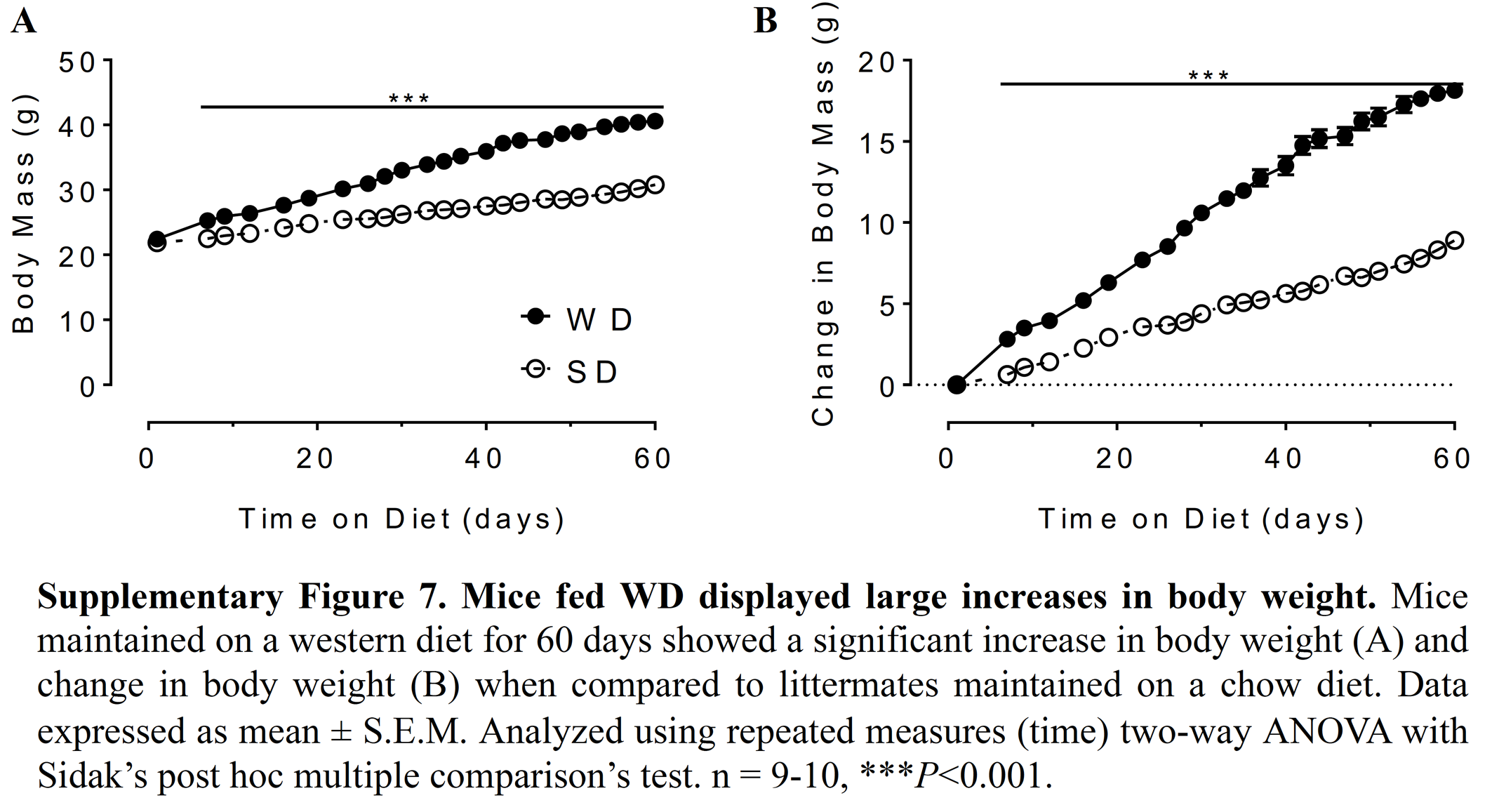

Supplement: Supplementary file 7 [file Image_7.TIF]
